# Rapid Detection of *Nocardia* by Next-Generation Sequencing

**DOI:** 10.3389/fcimb.2020.00013

**Published:** 2020-02-18

**Authors:** Shan-Shan Weng, Han-Yue Zhang, Jing-Wen Ai, Yan Gao, Yuan-Yuan Liu, Bin Xu, Wen-Hong Zhang

**Affiliations:** Department of Infectious Diseases, Huashan Hospital of Fudan University, Shanghai, China

**Keywords:** nocardiosis, next-generation sequencing, culture, diagnose, rapid detection

## Abstract

In this original study, we retrospectively reviewed the cases of nocardiosis diagnosed through culture and next-generation sequencing (NGS) methods between 2014 and 2018 in Huashan Hospital and found out that the latter way can not only improve the detection rate of *Nocardia* spp. but also greatly reduce the turnaround time. In addition, by comparing nocardiosis and non-nocardiosis patients both of whose samples had *Nocardia* spp. detected by NGS, we found that *Nocardia*'s specific reads ranking among top two might be a satisfactory cutoff value for clinical diagnosis of the disease. Our study introduced the promising value of the NGS method in the rapid diagnosis of nocardiosis.

## Introduction

Nocardiosis, caused by gram-positive aerobic actinomycetes in the genus *Nocardia*, is typically regarded as an opportunistic infection (Sorrell et al., 2010). *Nocardia* species are found worldwide in environments, and at least 33 of the total 80 more species have the ability to cause localized or systemic suppurative disease in human beings (Brown-Elliott et al., 2006). However, management of this rare infection is challenging, with mortality rates ranging from 20 o 30% in patients with disseminated infection, and up to 50% if there is central nervous system (CNS) involvement (Rouzaud et al., 2018).

Up to now, a definitive diagnosis of nocardiosis still relies on the isolation and identification of the organism from an infected site, which can take days to weeks. Molecular techniques such as 16S rRNA–based polymerase chain reaction (PCR) have also been used for rapid detection of *Nocardia* with high specificity and sensitivity (Couble et al., 2005), but such a technique requires the pre-considerations of nocardiosis by clinicians.

A previous study has reported that the next-generation sequencing (NGS) method could assist clinical decision making with minimized turnaround time as well as satisfying diagnostic performance, and also has the ability to identify nocardiosis in addition to traditional culture (Miao et al., 2018). Since January 2017, Huashan Hospital has gradually introduced NGS into the clinical approach to suspected infectious diseases with the aim of further increasing the chance of detecting pathogenic microorganisms. Here we retrospectively studied reported cases of nocardiosis identified through either NGS or conventional methods.

## Methods

### Study Design

We conducted a retrospective review of patients diagnosed with nocardiosis in Huashan Hospital (a tertiary hospital in Shanghai, China) from September 1st, 2014, to September 1st, 2018. During the time period, a total of 30 patients were diagnosed. Four outpatients and one patient referred from another hospital were excluded because of deficient evidence for definite diagnosis. Four other patients were diagnosed with pulmonary nocardiosis based on histopathology or 16S rRNA–based PCR results and were therefore excluded in our study. Ultimately, 21 cases of nocardiosis were enrolled in this retrospective study. The samples collected from these patients were separated into the following groups:

1. NGS-positive group (NG): *Nocardia* spp. detected in clinical samples by NGS between January 2017 and August 2018, with clinical and/or radiological signs indicating a diagnosis of nocardiosis regardless of the culture results of the same sample site.
2. Culture-positive group (CG): Samples reported culture positive for *Nocardia* spp. between 2014 and 2018, regardless of the synchronous NGS results.

The demographic data and clinical data of the patients were recorded (Table 1, Table S1). The NGS data including the specific species of *Nocardia*, number of detected reads, proportion, and ranking of the species were also recorded (Table S4). The turnaround time of each sample in the NG group (Table S6) and CG group (Table S7) were noted separately.

**Table 1:** Main characteristics of the nocardiosis population.

**Characteristics**	**Total (21)**
Age at diagnosis	49.5 ± 16.2
Male sex	10 (47.6%)
Immunocompromised state	14 (66.7%)
Suspicion for nocardiosis	4 (19.0%)
Antibiotic treatment before sampling	19 (90.5%)
SXT prophylaxis	1 (4.8%)
Culture positive	16 (76.2%)
Site of infection	
Systemic	9 (42.9%)
Pulmonary	7 (33.3%)
Central nervous system	4 (19.0%)
Cutaneous	1 (4.8%)

Besides, to evaluate the possibility of false positive detection of *Nocardia* by NGS, we further reviewed samples that underwent NGS testing during the same time period and defined those with *Nocardia* spp. that were detected but contradicted with the patient's final diagnosis as being in the non-nocardiosis group (NN). The NGS data as well as the clinical data of the NN group were also recorded (Table S5).

### Next-Generation Sequencing

#### Sample Processing and Library Construction

A 300 μl sample of cerebrospinal fluid (CSF), bronchoalveolar lavage fluid (BALF), tissue, sputum, etc. were collected in DNase/RNase tubes for the identification of potential pathogens. A common issue in metagenomic sequencing is the introduction of contaminating microbial nucleic acid during sample preparation. The potential contaminating source includes PCR reagents, nucleic acid extraction kits, human skin, as well as environment. In order to control the effect of contamination, a negative control was prepared in parallel and sequenced in the same run. Before nucleic acid extraction, the sputum was liquefied.

DNA and total RNA were extracted with a TIANamp Micro DNA Kit (DP316, TIANGEN BIOTECH, Beijing, China) and a QIAamp Viral RNA Mini Kit (52906, Qiagen, China) following the manufacturers' respective operational manuals. The RNA was reverse-transcribed and synthesized to double-stranded complementary DNA (ds cDNA) with a SuperScript II Reverse Transcription Kit (18064-014, Invitrogen, China). The DNA/cDNA from other samples was fragmented using a Bioruptor Pico instrument to generate 200–300 bp fragments (Bioruptor Pico protocols). Then, the libraries were constructed as follows: first, the DNA fragments were subjected to end-repair and added A-tailing in one tube; subsequently, the resulting DNA was ligated with bubble-adapters which contained a barcode sequence and then amplified by PCR method. Quality control was carried out using a bioanalyzer (Agilent 2100, Agilent Technologies, Santa Clara, CA, USA) to assess the DNA concentration and fragment size. Qualified libraries were pooled together to make a single-strand DNA circle (ssDNA circle) and then generate DNA nanoballs (DNBs) by rolling circle replication (RCA). The final DNBs were loaded into a sequence chip and sequenced on a BGISEQ platform using 50/100 bp single-end sequencing.

### Bioinformatics Analysis

All raw reads were quality-filtered using a made-in-house program, including filtering adapter contamination and low-quality and low-complexity reads. Next, the clean reads after quality filtering were mapped to a human reference database including hg38 and Yanhuang genome sequence using Burrows–Wheeler Alignment (Version: 0.7.10). The remaining reads were aligned to the non-redundant bacterial, virus, fungal, and parasite databases using Burrows–Wheeler Alignment (Version: 0.7.10). The mapped data were processed for advanced data analysis.

We downloaded all the reference genomes from public databases, such as NCBI (https://www.ncbi.nlm.nih.gov/genome). Currently, our databases contain 4,152 whole genome sequences of viral taxa, 3,446 bacterial genomes or scaffolds, 206 fungi related to human infection, and 140 parasites associated with human diseases. The depth and coverage of each species were calculated with the SoapCoverage software from the SOAP website (https://github.com/sunhappy2019/soap.coverage).

The parameter values were normalized according the data size, which is 8 million reads for sputum and BALF and 20 million reads for other samples. The detected species that existed in the suspected background database or/and was also detected in the negative control sample was filtered, if reaching the threshold.

## Results

Ultimately, 21 cases of nocardiosis were enrolled in this retrospective study (Table 1). Of the 21 patients, 9 (42.9%) had disseminated infection, 7 (33.3%) had pulmonary infection, 4 (19.0%) had CNS infection, and 1 (4.8%) had cutaneous infection. Fourteen (66.7%) were immunocompromised (undergoing glucocorticoid therapy or were organ transplant recipients) or had other underlying diseases such as tuberculosis.

**Figure 1:**
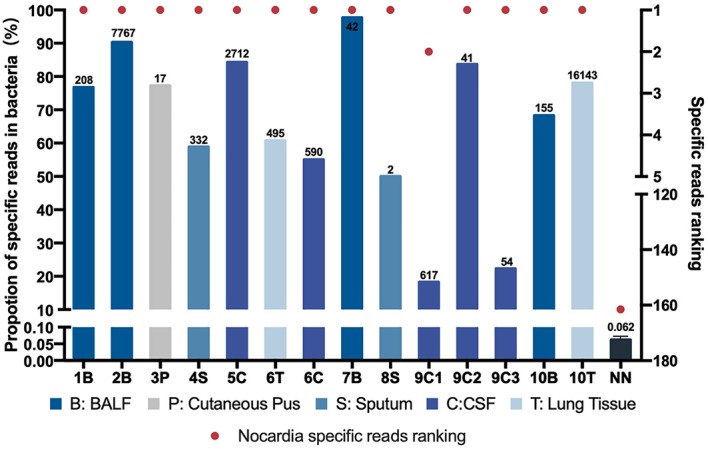
*Nocardia* specific reads and their ranking in next-generation sequencing (NGS)–positive (NG) and non-nocardiosis (NN) group patients. Data of 14 samples from 10 patients in the NG group are presented respectively. The data of the NN group are presented as medians. Each red point indicates the ranking of the *Nocardia* spp. detected, each length of the bar indicates the proportion rate, and the numbers on the bars indicate the specific reads. Different colors represent different sample types. B, BALF; P, cutaneous pus; S, sputum; C, CSF.

Among 21 patients, 25 samples were collected (multiple specimens were collected from 3 patients). Fourteen NGS-positive samples were classified into the NG group, and 11 culture-positive-only samples and 5 culture- and NGS-positive samples were classified into the CG group (Table 2). Besides, 55 samples were included in the NN group (Table S5). The NG group consisted of five CSF, four BALF, two sputum, two lung tissue, and one cutaneous pus, while the CG group consisted of three blood, three CSF, three BALF, three cutaneous pus, two sputum, one urine, and one pleural effusion (Figure 1, Table S3).

**Table 2:** Group of 25 samples from 21 nocardiosis patients according to NGS and culture results.

	**NGS not performed**	**NGS performed**		**Total**
		**NGS** **positive**	**NGS** **negative**	
Culture positive	11	5	0	16 (CG)
Culture negative	Unknown	9	Unknown	/
Total	/	14 (NG)	/	/

*NGS, next-generation sequencing; CG, culture-positive group; NG, NGS-positive group*.

All 14 samples in the NG group underwent culture simultaneously, and five reported positive results. Two of the samples had positive results on the first culture. Two other samples had positive results on the second culture. Another one had undergone a total of 10 cultures before finally obtaining a positive-culture result for *Nocardia* spp. (Table S6). The NGS data showed that all 14 samples' *Nocardia* spp. specific reads had ranked in the top two among all microbes, and 13 (92.9%) of the samples ranked at the top. In the NN group, however, the *Nocardia* spp. specific read ranking of the 55 samples ranged from 15 to 341, with an average of 163 (Figure 1, Table S5). In addition, the proportions of *Nocardia* specific reads in all identified microbe sequences ranged from 18.2 to 90.2% in the NG group compared to an average of 0.06% in the NN group. Classified by specimen types, the average proportion of *Nocardia* specific reads in BALF (83.3%) and lung tissue (69.4%) was higher than that of sputum (54.4%) and CSF samples (52.7%). Six different species of *Nocardia* were identified through NGS—*Nocardia terpenica, Nocardia otitidiscaviarum, Nocardia farcinica, Nocardia cyriacigeorgica, Nocardia brasiliensis*, and *Nocardia africana* (Table S4). Strains identified through the culture method, however, were not classified into specific species.

**Figure 2:**
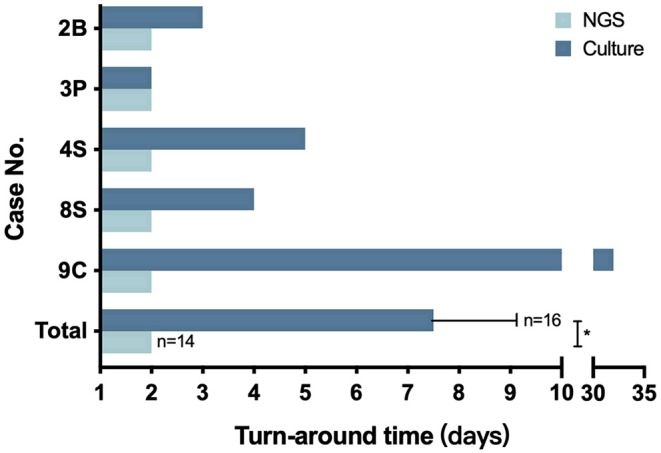
The turnaround time of NGS and culture. The number in the Y axis represents the patient number in the NG group. The bottom pair is presented as means with standard deviation. *P = 0.0054.

As for the CG group, the turnaround time of culture ranged from 2 to 32 days with an average time of 7.5 ± 1.92 days, which is significantly higher than the 48 h per sample of the NG group (*P* = 0.0054) (Figure 2).

## Discussion

Up to now, culture is still the most commonly used diagnostic method for nocardiosis in clinical practice. According to a previous report, it usually takes about 2 to 7 days for the cultures of *Nocardia* to be positive, sometimes prolonged to at least 2 weeks for slow-growing species (Patricia et al., 2012). Thus, in clinical settings, delay in establishing the diagnosis is common due to the non-specific and diverse clinical presentation of the disease and the time consumption in cultivating *Nocardia*. In fact, the recorded mean time from the development of symptoms to diagnosis has, in different studies, ranged from 42 days to 12 months (Georghiou and Blacklock, 1992; Martínez Tomás et al., 2007), which may lead to poor clinical outcomes in some patient groups (Simpson et al., 1981; Uttamchandani et al., 1994).

Due to the continuous development of sequencing technology, we have been able to conduct rapid detection of clinical specimens for patients and thus diagnosed some of the patients with nocardiosis, most of which were unexpected. Therefore, we retrospectively analyzed these cases of nocardiosis and tried to analyze the application value of the NGS testing method in diagnosing this easily missed disease.

In our study, 14 samples underwent both culture and NGS testing. All 14 samples were reported to be NGS-positive for *Nocardia* spp., while only 5 obtained culture-positive results. Nine samples failed the cultivation, and repeated cultures during hospitalization did not yield positive results either (Table S6). As previously reported, the difficulty of cultivating may be due to the strict requirements of some *Nocardia* spp. on the culture conditions and its slow growth ability. On the other hand, most patients in our study had been treated with antibiotics before sampling (Table 1, Table S2), which might also reduce the detection rate. This result suggested that compared with conventional methods, NGS was more efficient and sensitive in detecting *Nocardia* spp. Besides, the turnaround time had also been significantly reduced to 2 days by NGS, compared with an average of 7.5 days by culture (Figure 2). Although the sensitivity and specificity of NGS need to be further tested by well-designed studies with larger sample sizes, we believe that this method could be a promising tool in clinical practice.

As for the antibiotic susceptibility, only 3 of the 16 CG samples had related records, while the rest had nothing recorded, which may be due to incomplete records. These three strains were collected from patients 8, 18, and 19, respectively, and the drug susceptibility results were, in order, sensitive to all commonly used drugs, sensitive to sulfamethoxazole (STX) and linezolid, and sensitive to linezolid and levofloxacin while resistant to STX. The treatment of these three patients was based on the results of drug susceptibility (Table S2). For patients diagnosed with NGS alone, there were no strains for the antibiotic susceptibility test, and thus, medications could not be chosen accordingly. However, since *Nocardia* could be identified to species through NGS, this information may lead to an accurate antibiotic treatment based on the reported common susceptibility patterns of each individual species (Brown-Elliott et al., 2006).

In the meanwhile, through further analysis of the cases in the NG group, we found that NGS testing might compensate for the low detection ability of conventional culture methods in certain sample types. In our study, NGS was conducted in samples of different kinds such as CSF, BALF, sputum, cutaneous pus, and lung tissue. The only cutaneous pus sample and two sputum samples were reported to be both culture- and NGS-positive. Of the four BALF samples, one was reported to be culture positive. For the two lung tissue samples, NGS detected numerous reads and a high proportion of *Nocardia* spp., while repeated cultures reported negative results. Similarly, for five CSF samples, only one was reported positive after 32 days of culture, while the other four failed to get culture-positive results. This result suggested that NGS might have good detection potential for *Nocardia* in lung tissue and CSF samples.

By comparing the NGS data of the NN group and the NG group, we further explored the possible cutoff values of nocardiosis. With the specific reads ranging from 2 to 16,143, all detected *Nocardia* species ranked among the top two in the overall specific read ranking of each sample in the NG group. However, in the NN group, the specific reads ranged from 0 to 8 with the rankings from 15 to 341. In the NG group, the proportions of *Nocardia* specific reads in all detected microbial sequences ranged from 18.2 to 90.2% among different types of samples, compared with an average of 0.062% in the NN group. Therefore, our study suggested that *Nocardia*'s specific reads ranking among the top two might be a satisfactory cutoff value for clinical diagnosis of the disease. Due to the limitations of this study, we may regard the value not as an absolute diagnostic threshold but as a reference for clinical diagnosis.

In conclusion, our study originally illustrated the application of NGS in diagnosing nocardiosis and found that it had a satisfactory diagnostic value compared to conventional methods. NGS could not only identify different *Nocardia* species but also greatly reduce the detection turnaround time and thus contribute to a timely clinical diagnosis and treatment. Besides, by comparing nocardiosis and non-nocardiosis patients both of whose samples had *Nocardia* spp. detected by NGS, we suggested that *Nocardia*'s specific reads ranking among the top two might be a satisfactory cutoff value for clinical diagnosis of the disease. However, with the limit of sample size, further investigations and evaluation are necessary. Up to now, the high cost and low accessibility of NGS still limit its utility in general practice. We hope that in the near future, this technology will serve the clinic more appropriately and benefit more patients.

## Data Availability Statement

The datasets generated for this article can be found in European Nucleotide Archive (ENA) using the accession number PRJEB34974 (https://www.ebi.ac.uk/ena/browser/view/PRJEB34974).

## Ethics Statement

The studies involving human participants were reviewed and approved by the ethics committee of Huashan Hospital (Shanghai, China). The patients/participants provided their written informed consent to participate in this study.

## Author Contributions

S-SW designed the study, collected and analyzed the data, wrote, and edited the manuscript. H-YZ collected and analyzed the data and wrote the manuscript. J-WA designed the study, collected the data, and edited the manuscript. YG, BX, and Y-YL collected the samples and diagnosed and treated all the patients. W-HZ designed the study and was in charge of the overall cohort management.

### Conflict of Interest

The authors declare that the research was conducted in the absence of any commercial or financial relationships that could be construed as a potential conflict of interest.
