# Supplementary material for: Rapid Detection of Nocardia by Next-Generation Sequencing
Source: Front Cell Infect Microbiol. 2020 Feb 18;10:13. doi: 10.3389/fcimb.2020.00013 (PMC7040243; doi:10.3389/fcimb.2020.00013)
Supplement: Supplementary file 1 [file Data_Sheet_1.pdf]

Supplementary Materials

Table S1. Characteristics of patients in NG group

Case No.	Gender	Age(years)	Medical history	Associated manifestations	Sites of infection
1	Male	38	None	Dizziness, headache, fever	Systemic (pulmonary,CNS)
2	Male	82	Drug eruption	None	Pulmonary
3	Female	68	None	Feverishness on dorsum of hand	Cutaneous
4	Female	42	Pemphigus (hormonotherapy)	Fever, coughing, chest tightness	Pulmonary
5	Male	84	Hormonotherapy	Headache, dizziness, fever, vomiting, weight loss	CNS
6	Male	42	Pemphigus (hormonotherapy)	Fever, chest pain	Pulmonary

7	Female	42	Pemphigus (hormonotherapy)	Fever, coughing, chest tightness	Pulmonary
8	Female	34	Evan syndrome (hormonotherapy)	Emptysis, dermal ulcer, knee pain	Systemic (pulmonary,cutaneous)
9	Male	53	Obsolete pulmonary tuberculosis, Bronchiectasis	Fever, coughing, unconsciousness, weight loss	Systemic (pulmonary,cutaneous, CNS)
10	Male	42	Cryptococcal meningitis, Latent pulmonary tuberculosis	Fever, headache, blurred vision, vomiting	Systemic (pulmonary,CNS)

Table S2. Treatment related information of patients in NG and CG group

Case No.*	Suspicion for nocardiosis	Treatment before diagnosis	Treatment after diagnosis	Status on discharge	Co-infection
1	No	Meropenem	Linezolid+meropenem	Improvement	Hepatitis B
2	No	Meropenem+voriconazoleg+fosfomycin	Imipenem and cilastatin sodium+amikacin	Improvement	Pulmonary aspergillosis
3	No	None	Ceftriaxone+amikacin	Improvement	None

4	No	Voriconazole+ moxifloxacin+caspofungin+posaconazole	Linezolid+meropenem	Improvement	None
5	No	Meropenem+vancomycin	Meropenem+sulfamethoxazole+fluconazole+ ciprofloxacin	Improvement	None
6	No	Cefoperazone sodium and sulbactam sodium+fluconazole	Meropenem+vancomycin	No progression	Pulmonary cryptococcosis; hepatitis B
7	No	None	Sulfamethoxazole	No progression	None
8	No	Cefazolin	Sulfamethoxazole	No progression	None
9	No	Meropenem+piperacillin sodium and tazobactam sodium+metronidazole	Linezolid+amikacin	Progression	Latent tuberculosis infection; hepatitis B; severe pneumonia caused by Klebsiella pneumonia
10	Yes	sulfamethoxazole+flucytosine+fluconazole	Linezolid+amikacin+ceftriaxone	Improvement	Cryptococcus meningitis

11	No	isoniazid+rifampicin+pyrazinamide+ethambutol+levofloxacin	Linezolid+amikacin+ceftriaxone+levofloxacin	Improvement	None
12	Yes	Linezolid+piperacillin sodium and tazobactam sodium	sulfamethoxazole+linezolid+penicillin	Improvement	None
13	Yes	Meropenem+linezolid	Linezolid+meropenem	Improvement	Hepatitis B
14	No	Isoniazid+rifampicin+pyrazinamide+ethambutol	Sulfamethoxazole+isoniazid+rifampicin+pyrazinamide+ethambutol	Improvement	Tuberculosis infection(possibly)
15	No	Not accessible	Sulfamethoxazole+ceftriaxone	Improvement	None
16	No	Isoniazid+rifampicin+pyrazinamide+ethambutol+linezolid+amikacin	Sulfamethoxazole+Meropenem+linezolid+amikacin	Improvement	None
17	No	Meropenem+ vancomycin	Sulfamethoxazole+Meropenem+linezolid	Improvement	None
18	No	Voriconazole	Sulfamethoxazole+ceftriaxone+linezolid	Improvement	None
19	Yes	Itraconazole	Linezolid+levofloxacin	Improvement	None
20	No	Voriconazole	Linezolid+imipenem and cilastatin sodium	Improvement	Penicillium marneffei infection

21	No	Moxifloxacin	Imipenem and Cilastatin Sodium+Amikacin	Improvement	None

*Case 1-10 belonged to NG group, case 11-21belonged to CG group.

Table S3. Sample types of all the included samples

Characteristics	CG samples (16)	NG samples (14)	NN samples (55)	Total (85)
Sample type				
Cerebrospinal fluid	3 (18.8%)	5 (35.7%)	39 (70.9%)	47 (55.3%)
Bronchoalveolar lavage fluid	3 (18.8%)	4 (28.6%)	10 (18.2%)	17 (20.0%)
Sputum	2 (12.5%)	2 (14.3%)	2 (3.6%)	6 (7.1%)
Lung tissue	0 (0%)	2 (14.3%)	0 (0%)	2 (2.4%)
Cutaneous pus	3 (18.8%)	1 (7.1%)	0 (0%)	4 (4.7%)
Skin tissue	0 (0%)	0 (0%)	4 (7.3%)	4 (4.7%)
Blood (Plasma for NGS)	3 (18.8%)	0 (0%)	0 (0%)	3 (3.5%)
Urine	1 (6.3%)	0 (0%)	0 (0%)	1 (1.2%)

Pleural effusion	1 (6.3%)	0 (0%)	0 (0%)	1 (1.2%)

Table S4. NGS data of patients in NG group

Case No.	Species	Sample type	Reads	Rank	Proportion Rate
1	*Nocardia farcinica*	BALF	208	1	0.76752768
2	*Nocardia farcinica*	BALF	7767	1	0.90313953
3	*Nocardia brasiliensis*	Cutaneous pus	17	1	0.77272727
4	*Nocardia brasiliensis*	Sputum	332	1	0.58865248
5	*Nocardia farcinica*	CSF	2712	1	0.84302145
6	*Nocardia africana*	Lung tissue	495	1	0.60736196
6	*Nocardia africana*	CSF	590	1	0.55037313
7	*Nocardia cyriacigeorgica*	BALF	42	1	0.97674419
8	*Nocardia brasiliensis*	Sputum	2	1	0.5
9	*Nocardia terpenica*	CSF	617	2	0.18254438
9	*Nocardia terpenica*	CSF	41	1	0.83673469
9	*Nocardia terpenica*	CSF	54	1	0.2231405
10	*Nocardia otitidiscaviarum*	BALF	155	1	0.68281938

10	*Nocardia otitidiscaviarum*	Lung tissue	16143	1	0.78072254

CSF: Cerebrospinal Fluid; BALF: Bronchoalveolar Lavage Fluid

Table S5. NGS data of patients in NN group

Case No.	Species	Sample type	Reads	Rank	Proportion Rate
22	Nocardia	Skin tissue	0	67	0.000822966
23	Nocardia	Skin tissue	0	15	0.010557634
24	Nocardia	Skin tissue	0	105	2.68351E-06
25	*Nocardia brasiliensis*	Skin tissue	8	248	0.000595166
26	Nocardia	Sputum	0	162	2.25644E-06
27	Nocardia	Sputum	0	93	1.77655E-05
28	Nocardia	BALF	0	78	6.96341E-05
29	Nocardia	BALF	0	98	1.95951E-05
30	Nocardia	BALF	0	115	5.42248E-05
31	Nocardia	BALF	0	212	5.71788E-05
32	Nocardia	BALF	0	164	1.96199E-05
33	Nocardia	BALF	0	81	6.99848E-05
34	Nocardia	BALF	0	189	2.65677E-05
35	Nocardia	BALF	0	177	5.30294E-06
36	Nocardia	BALF	0	215	0.000245557
37	Nocardia	BALF	0	142	0.000168214
38	Nocardia	CSF	0	147	0.000276185
39	Nocardia	CSF	0	78	0.001079721
40	Nocardia	CSF	0	322	0.000789636

41	Nocardia	CSF	0	33	0.002545873
42	Nocardia	CSF	0	132	0.001434436
43	Nocardia	CSF	0	256	0.000769133
44	Nocardia	CSF	0	341	0.000460184
45	Nocardia	CSF	0	185	0.000570775
46	Nocardia	CSF	0	101	0.001220753
47	Nocardia	CSF	0	91	0.000460373
48	Nocardia	CSF	0	163	0.000190162
49	Nocardia	CSF	0	71	0.000120636
50	Nocardia	CSF	0	309	0.00021545
51	Nocardia	CSF	0	152	0.000360039
52	Nocardia	CSF	0	340	0.000538973
53	Nocardia	CSF	0	184	0.00053068
54	Nocardia	CSF	0	94	0.001268534
55	Nocardia	CSF	0	272	0.00040867
56	Nocardia	CSF	0	168	5.0688E-05
57	Nocardia	CSF	0	268	0.000610783
58	Nocardia	CSF	0	91	0.0003985
59	Nocardia	CSF	0	137	0.000483494
60	Nocardia	CSF	0	269	0.000135084
61	Nocardia	CSF	0	195	0.00028278
62	Nocardia	CSF	0	224	0.000380698

63	Nocardia	CSF	0	181	8.69734E-05
64	Nocardia	CSF	0	330	0.000348292
65	*Nocardia terpenica*	CSF	2	201	0.000424383
66	*Nocardia asteroides*	CSF	2	177	0.000670529
67	*Nocardia paucivorans*	CSF	2	161	0.00022756
68	*Nocardia africana*	CSF	1	110	0.000237176
69	*Nocardia asiatica*	CSF	1	95	0.00068315
70	*Nocardia nova*	CSF	2	124	0.00035189
71	*Nocardia concava*	CSF	5	136	0.000620607
72	*Nocardia higoensis*	CSF	4	125	0.00017582
73	*Nocardia asiatica*	CSF	4	172	0.00059175
74	*Nocardia terpenica*	CSF	1	145	0.000497218
75	*Nocardia higoensis*	CSF	1	48	0.000430326
76	*Nocardia arizonensis*	CSF	8	177	0.000203584

CSF: Cerebrospinal Fluid; BALF: Bronchoalveolar Lavage Fluid

frontiers

Table S6. Turn-around time of NGS and culture of patients in NG group

Case No.	Species	Sample type	Turn-around time of NGS (days)	Turn-around ti[illegible] culture (days)
1	Nocardia farcinica	BALF	2	\
2	Nocardia farcinica	BALF	2	4
3	Nocardia brasiliensis	Cutaneous pus	2	2
4	Nocardia brasiliensis	Sputum	2	5
5	Nocardia farcinica	CSF	2	\
6	Nocardia africana	CSF	2	\
6	Nocardia africana	Lung tissue	2	\
7	Nocardia cyriacigeorgica	BALF	2	\
8	Nocardia brasiliensis	Sputum	2	4
9	Nocardia terpenica	CSF	2	\
9	Nocardia terpenica	CSF	2	32
9	Nocardia terpenica	CSF	2	\
10	Nocardia otitidiscaviarum	BALF	2	\
10	Nocardia otitidiscaviarum	Lung tissue	2	\

* For samples which finally obtained culture-positive results, times of culture referred to the total culture times until the final culture-positive result came out. For samples which failed to obtain a culture-positive result, times of culture referred to total culture times during the hospitalization.

** BALF and sputum were included.

*** Lung tissue, BALF and sputum were included.

Table S7. Turn-around time of culture of patients in CG group and part of the patients in NG group

Case No.*	Species	Sample type	Turn-around time (days)
4	Nocardia brasiliensis	Sputum	5
8	Nocardia brasiliensis	Sputum	4
3	Nocardia brasiliensis	Cutaneous pus	2
9	Nocardia terpenica	CSF	32
2	Nocardia farcinica	BALF	4
11	Nocardia	Urine	5
12	Nocardia	Pus	4
13	Nocardia	Pus	3
14	Nocardia	Pleural Drainage	6
15	Nocardia	CSF	6
16	Nocardia	CSF	4
17	Nocardia	Blood	7
18	Nocardia	Blood	10
19	Nocardia	Blood	14
20	Nocardia	BALF	6
21	Nocardia	BALF	8

CSF: Cerebrospinal Fluid; BALF: Bronchoalveolar Lavage Fluid

*Case 4,8,3,9,2 belonged to NG group, case 11-21 belonged to CG group.
